# Psychological distress, loneliness, alcohol use and suicidality in New Zealanders with mental illness during a strict COVID-19 lockdown

**DOI:** 10.1177/00048674211034317

**Published:** 2021-07-27

**Authors:** Caroline Bell, Jonathan Williman, Ben Beaglehole, James Stanley, Matthew Jenkins, Philip Gendall, Charlene Rapsey, Susanna Every-Palmer

**Affiliations:** 1 Department of Psychological Medicine, University of Otago, Christchurch, New Zealand; 2 Department of Population Health, University of Otago, Christchurch, New Zealand; 3 Department of Public Health, University of Otago, Wellington, New Zealand; 4 Department of Psychological Medicine, University of Otago, Wellington, New Zealand; 5 Department of Psychological Medicine, University of Otago, Dunedin, New Zealand

**Keywords:** Mental illness, well-being, COVID-19, lockdown, suicide, anxiety

## Abstract

**Introduction:**

People with pre-existing mental health conditions may have been disproportionally impacted by the COVID-19 pandemic and associated public health restrictions. In this study, we compared psychological outcomes, experiences and sources of stress over the pandemic lockdown in New Zealanders with and without a previous diagnosis of mental illness.

**Methods:**

Two online surveys were conducted in New Zealand over the level 4 lockdown in April 2020 measuring psychological distress, anxiety, well-being, suicidality, alcohol use and subjective experiences. They included 3389 participants, of whom 18.4% reported having been previously diagnosed with a mental illness.

**Results:**

During the lockdown, people previously diagnosed with a mental illness had about twice the risk of reporting moderate-high levels of psychological distress (K10 ⩾ 12), at least moderate levels of anxiety (GAD-7 ⩾ 10) and poor well-being (WHO-5 ⩽ 12). They reported increased alcohol use and were about four times as likely to have experienced suicidal thoughts with 3% reporting having made a suicide attempt over the lockdown period. They reported less satisfaction with, and poorer relationships with people in their ‘bubble’, reduced social contacts and greater loneliness. They also reported higher levels of health and financial concerns.

**Conclusion:**

During the COVID-19 lockdown in New Zealand, people with a previous diagnosis of a mental illness were at increased risk of detrimental psychological outcomes. This highlights the importance of recognising this and the challenges people face in pandemics.

There is increasing recognition of the psychological impacts of the COVID-19 pandemic (Ghebreyesus, 2020) relating to the complex mix of the fear of infection and the social and economic effects of public health restrictions (Holmes et al., 2020). Given that population studies find individuals with a previous diagnosis of a mental disorder are at greater risk of onset of a subsequent episode (Kessler et al., 2007), it is important to consider how the pandemic context may influence risk. Several commentaries, published early in the pandemic, presciently highlighted the importance of understanding these impacts on those with pre-existing mental health disorders (Druss, 2020; Yao et al., 2020). Specifically, these authors noted that the disruption to social and healthcare networks, and the potentially disproportionate financial and employment ramifications, may present particular challenges for this group. Subsequent general population studies reported that a pre-existing mental health condition is an independent risk factor for detrimental psychological outcomes during the pandemic (Every-Palmer et al., 2020; O’Connor et al., 2020; Robillard et al., 2020).

Literature specifically exploring the psychological impacts of the pandemic in people with mental illness has produced mixed findings. Early in the pandemic, cross-sectional studies from China (Hao et al., 2020), Italy (Iasevoli et al., 2020), Australia (Van Rheenen et al., 2020), the United States and Canada (Asmundson et al., 2020), and Denmark (Rohde et al., 2020) consistently reported higher rates of anxiety, depression and distress in people with a mental health disorder compared to those without such a diagnosis. However, studies from longitudinal samples reported more nuanced findings. A study from the Netherlands of three case–control cohorts reported higher rates of psychological symptoms in those with a mental illness compared to those without, both before and since COVID-19, but no greater increase in symptoms over the pandemic (Pan et al., 2021). An American study similarly reported a universal pattern of distress (i.e. similar in those with and without a previous diagnosis of mental illness), which increased as the pandemic first emerged, with recovery over the following 2 months (Daly and Robinson, 2020). Recent studies have reported that those with a previous mental health disorder have an increased risk of post-traumatic stress disorder (PTSD).

In addition to the psychological impacts, studies have also consistently reported that mental health disorders, particularly severe mental illnesses, are associated with an increased risk of COVID-19 infection and of adverse morbidity and mortality from this (Taquet et al., 2021; Yang et al., 2020). This is likely to be because of medical and socioeconomic factors, the high frequency of medical comorbidities related to poor COVID-19 outcomes, high rates of smoking, living environments and ability to access care when ill. There is therefore a strong ethical case for this group to be prioritised in vaccination strategies (implemented in some countries such as the United Kingdom), and for those providing care to provide clear information of the benefits and risk of vaccination (Mazereel et al., 2021).

The aim of our study was to add to the literature by comparing psychological outcomes, experiences and sources of stress over the pandemic lockdown in New Zealand in people with and without a previous diagnosis of mental illness. It is noteworthy that the New Zealand lockdown was particularly stringent. In early April 2020, NZ was scoring 96/100 on the Oxford University COVID-19 stringency index (a composite measure for comparing the restrictiveness of responses), and this was the highest score of any World Bank high-income country. Understanding the stressors and the mental health and well-being of people with mental illness is important to inform their needs and appropriate psychosocial supports. This knowledge may be particularly pertinent in the global environment with the implementation of further lockdowns and restrictions, and to inform planning for further pandemics.

## Methods

Our study involved an online survey of adult New Zealanders. The survey questions were designed to assess levels of psychological distress, anxiety, well-being, suicidality, alcohol consumption and family relationships during the COVID-19 lockdown using validated measures with benchmark comparisons where possible. We aimed to recruit a sample that represented the New Zealand adult population. We recruited participants via two methods. First, we a used commercial survey platform (Dynata) which applied target participation quotas by age, sex and ethnicity (Every-Palmer et al., 2020).Second, we invited the participation of New Zealanders who had previously been randomly selected by the NZ Ministries of Health and Justice (identified using stratified multi-stage area sampling strategies) to participate in national data collection and who had consented to further contact. We have already reported some of the population outcomes for the first method (Every-Palmer et al., 2020).

### Survey

The survey was fielded using the Qualtrics platform between 15 April and 27 April 2020, during the New Zealand lockdown. It could be completed on a mobile phone, tablet or computer and took approximately 15 minutes (see Every-Palmer et al., 2020, for more details). The survey questions are available as a supplementary file (Supplementary file 1) and a detailed description of survey items and construction of the questionnaire has previously been published (Every-Palmer et al., 2020).

### Participants

Participants were eligible to complete the survey if they were aged 18 years or older at the time of the level 4 lockdown.

Data from participants recruited from the Dynata and NZ Ministry of Justice and Ministry of Health sampling frames were combined. Due to differences in sampling methodology, survey weights were not applied as the analysis was focused on identifying relationships between variables, rather than deriving national estimates of population characteristics. However, sensitivity analysis (Supplementary file 2) was performed to confirm broad comparability of risk ratios across the two data sets. Data were broadly comparable, which allowed them to be combined. We achieved responses from a total of 3389 respondents; 624 (18.4%) who reported they had previously been diagnosed with a mental illness and 2765 (82.6%) who did not report a past history of mental illness.

### Measures

#### Mental illness diagnosis

Participants were asked if they had been diagnosed with a mental illness by a doctor or psychologist and to identify their diagnosis from options which included depression, bipolar disorder, anxiety disorder, psychotic disorder, personality disorder, alcohol or drug disorder, or other, from which multiple options were allowed. They could also answer ‘don’t know’ or ‘prefer not to say’. Those who indicated they had a mental illness were asked how their mental health had been during the lockdown, with the six options available being: much better than usual, better than usual, the same as usual, worse than usual, much worse than usual or prefer not to say.

#### Demographic factors lockdown experiences and stressors

We asked about demographic and pre-lockdown factors including age, gender, ethnicity, socio-economic status (education and household income), employment, smoking and alcohol consumption. In addition, we asked about health vulnerability (i.e. over the previous 5 years, had they had a medical condition that might make them more vulnerable to COVID-19 such as heart disease, chronic obstructive pulmonary disease [COPD; difficulty breathing], weakened immunity or cancer). We also asked about subjective prior exposure to trauma from a list including neglect, physical or sexual abuse, natural disaster, serious physical injury or illness or ‘other’.

Objective and subjective lockdown experiences were assessed by questions about living circumstances, relationships and connections with others, workload (increased workload, reduced paid hours of work, termination of employment), change in alcohol use (i.e. how many standard drinks they consumed on an average 7-day period before the lockdown and how many standard drinks they had consumed over the previous 7 days), COVID-19 exposure and concerns about risk of infection. Participants were asked to identify what main sources of stress were (uncertainty about their health or that of a family member; finances; employment; the wider consequences of COVID-19). They were also asked about suicidal thoughts, plans and attempts over the previous 12 months before, and over the lockdown.

#### Psychometric measures

The survey contained three standardised measures – the Kessler Psychological Distress Scale (K10) (Kessler et al., 2003), the Generalised Anxiety Disorder Assessment Scale (GAD-7) (Spitzer et al., 2006) and the World Health Organization Well-Being Index (WHO-5) (Topp et al., 2015). The K10 is a 10-item scale measuring non-specific symptoms of anxiety and depression over the previous 4 weeks. Scores are reported in a 0–40 range to align with reporting in the New Zealand Health Survey (Ministry of Health New Zealand, 2020), with people scoring 12 or higher having moderate to high distress. The GAD-7 measures anxiety symptoms with respondents indicating how much they have been bothered by each of seven symptoms over the last 2 weeks, on a 4-point scale ranging from ‘not at all’ to ‘nearly every day’. Scores range from 0 to 21 with scores of 10 and higher indicating at least moderate anxiety. The WHO-5 is one of the most widely used scales for assessing subjective psychological well-being (Topp et al., 2015). It contains five positively phrased items, with respondents rating each statement for the last 2 weeks. Scores range from 0 to 25 with scores of 12 and under indicating poor well-being.

### Ethical approval

The study was approved by the University of Otago Human Ethics Committee (approval code F20/003) and underwent Māori consultation with the Ngāi Tahu Research Committee.

### Statistical analyses

Participants’ demographics, socioeconomic characteristics and health histories were summarised using counts and percentages. The proportion of participants reporting poor outcomes on each of the K10, GAD-7 and WHO-5 psychological measures, suicidality (thoughts, plans, attempts) and increase in alcohol consumption, was determined by previous diagnosis of mental illness or not category, and associations assessed using chi-square tests. Differences between previous diagnosis of a mental illness or not categories were quantified as risk ratios with 95% confidence intervals (CIs), calculated using a series of unadjusted and confounder adjusted Poisson generalised linear regression models with robust ‘sandwich’ standard errors (Zou, 2004). Participants’ experiences of lockdown were summarised by previous diagnosis of mental illness or not category as counts and percentages, and groupwise differences assessed using chi-square or Fisher’s exact tests. Analysis was performed using the R programming language and environment (R version 4.0.3).

## Results

### Demographic and socioeconomic characteristics

Table 1 compares the demographic profile of participants reporting a diagnosis of mental illness compared with those with no diagnosis of mental illness. Approximately two-thirds of those with a mental illness diagnosis were female. While most participants did not report pre-existing vulnerabilities to COVID-19 (e.g. being immunocompromised), twice as many people with mental health diagnoses considered themselves vulnerable (13.3% compared with 6.9%). This group also reported higher rates of smoking (21.4% compared to 14.4%). A history of exposure to previous trauma was more common (37.8% compared to 27.8%), with the most common trauma being exposure to a natural disaster.

**Table 1.** Demographic characteristics of respondents with and without a previous diagnosis of mental illness.

Characteristic	Previous diagnosis mental illness (*N* = 624)	No previous mental illness diagnosis (*N* = 2765)
Gender		
Male	32.1% (200)	45.6% (1,261)
Female	67.9% (424)	54.4% (1,504)
Age		
15–24	11.1% (70)	8.6% (238)
25–34	19.6% (124)	16.0% (445)
35–44	22.0% (139)	15.6% (434)
45–54	22.2% (140)	16.5% (459)
55–64	15.3% (97)	17.6% (488)
65+	9.8% (62)	25.7% (714)
Ethnicity (prioritised)		
Māori	20.4% (129)	17.1% (474)
Pacific	2.2% (14)	4.6% (128)
Asian	3.0% (19)	10.6% (294)
European/Other^a^	74.4% (470)	67.7% (1,882)
Education		
No formal qualification	11.1% (70)	10.8% (300)
High school	20.9% (132)	29.3% (814)
Certificate or diploma	29.3% (185)	24.1% (670)
Bachelor’s degree	22.5% (142)	21.1% (587)
Post-graduate	16.3% (103)	14.7% (407)
Income		
NZ $30,000 or less	39.6% (249)	31.0% (858)
NZ $30,001–NZ $70,000	32.6% (206)	34.7% (959)
NZ $70,001–NZ $100,000	11.0% (69)	12.7% (351)
NZ $100,001–NZ $150,000	6.7% (42)	7.0% (194)
NZ $150,001 or more	3.3% (21)	3.8% (104)
Prefer not to say	6.8% (43)	10.8% (300)
In workforce	62.0% (392)	64.9% (1802)
Potential vulnerability		
Health vulnerability	13.3% (84)	6.9% (191)
Smoker	21.4% (135)	14.4% (399)
History of previous exposure to trauma	37.8% (239)	27.8% (772)

a People of non-European ethnicity represent approximately 2% of the individuals in this group.

Of those with a previous diagnosis of mental illness, 79.0% (499) reported depression, 49.5% (313) an anxiety disorder, 5.2% (33) bipolar disorder, 5.1% (32) an alcohol or drug disorder, 4.1% (26) a personality disorder, 3.2% (20) a psychotic disorder and 10.4% (66) ‘other’.

### Psychological distress, anxiety, well-being, suicidality and alcohol use

As shown in Table 2, respondents with a previous diagnosis of mental illness were at about twice the risk of reporting moderate to high psychological distress (K10 ⩾ 12), anxiety (GAD-7 ⩾ 10) and poor well-being (WHO-5 ⩽ 12) not <13 as before. They were at three to four times the risk of having experienced suicidal thoughts and plans, respectively. Three percent reported having made a suicide attempt over the lockdown period. There was a small difference between those with and without previous diagnosis of mental illness in terms of increased alcohol use, with 30% and 24% of respondents, respectively, reporting this.

**Table 2.** Unadjusted and adjusted rates of psychological distress, anxiety, poor well-being and increase in suicidality and alcohol use.

	% (Number)	Risk ratio (95% confidence interval)	Adjusted risk ratio (95% confidence interval)a	Adjusted risk ratio (95% confidence interval)b
K10 ⩾ 12				
Previous diagnosis of mental illness	42.9% (271)	2.32[2.06, 2.61]	2.03[1.80, 2.29]	1.92[1.69, 2.17]
No previous diagnosis of mental illness	18.5% (513)	1.00	1.00	1.00
GAD-7 ⩾ 10				
Previous diagnosis of mental illness	25.2% (159)	2.97[2.47, 3.56]	2.59[2.15, 3.11]	2.37[1.96, 2.87]
No previous diagnosis of mental illness	8.5% (235)	1.00	1.00	1.00
WHO-5 ⩽ 12				
Previous diagnosis of mental illness	66.3% (417)	1.66[1.54, 1.78]	2.44[2.02, 2.94]	1.50[1.36, 1.66]
No previous diagnosis of mental illness	40.0% (1105)	1.00	1.00	1.00
Suicidal thoughts				
Previous diagnosis of mental illness	12.0% (73)	4.97[3.60, 6.85]	4.40[3.10, 6.24]	3.57[2.51, 5.09]
No previous diagnosis of mental illness	2.4% (66)	1.00		
Suicidal plans				
Previous diagnosis of mental illness	3.9% (24)	4.26[2.45, 7.41]	3.70[1.95, 7.03]	3.07[1.59, 5.94]
No previous diagnosis of mental illness	0.9% (25)	1.00		
Suicide attempts				
Previous diagnosis of mental illness	3.2% (20)	4.40[2.38, 8.14]	4.69[2.28, 9.95]	3.77[1.93, 7.37]
No previous diagnosis of mental illness	0.7% (20)	1.00		
Increased alcohol consumption				
Previous diagnosis of mental illness	30.2% (190)	1.25[1.09, 1.43]	1.12[0.98, 1.29]	1.16[1.01, 1.34]
No previous diagnosis of mental illness	24.2% (671)	1.00	1.00	1.00

GAD-7: Generalised Anxiety Disorder; WHO-5: World Health Organization; K10: Kessler Psychological Distress Scale.

a Adjusted for age, gender and ethnicity.

b Adjusted for age, gender, ethnicity, income, smoking status, employment, living alone, health vulnerability and prior exposure to a traumatic event.

Observed differences in outcomes between those with versus without a previous diagnosis of mental illness were somewhat reduced but persisted after controlling for potential confounders (age, gender, ethnicity, socioeconomic status, employment, living alone, health vulnerability and exposure to previous trauma).

### Living circumstances, connections, workload and COVID-19 testing

There was no evidence of a difference between respondents with and without a previous diagnosis of a mental illness in terms of their living circumstances (see Table 3). However, those with a mental illness reported less satisfaction with, and poorer relationships with people in their ‘bubble’ (a term coined by the NZ government and widely adopted to mean those you had close contact with over the lockdown). They also reported higher rates of reduced social contacts with family and friends outside of their bubble (which included contact by video link, telephone, email or letter). They were more likely to feel lonely or isolated (41% compared with 26%).

**Table 3.** Living circumstances, social connections, workload and COVID-19 testing.

Living circumstances, social connections, work demand and COVID-19 testing	Previous diagnosis of mental illness	No previous diagnosis of mental illness	*p*
Living circumstances			
Living situation			
Living alone	15.8% (100)	15.1% (418)	0.124
With one adult	29.1% (184)	33.1% (917)	
With other adults	22.5% (142)	19.0% (528)	
With children	32.6% (206)	32.8% (911)	
Satisfaction with ‘bubble’			
Extremely dissatisfied	4.9% (31)	4.1% (115)	<0.001
Dissatisfied	5.4% (34)	1.8% (49)	
Neither satisfied nor dissatisfied	10.8% (68)	8.5% (236)	
Satisfied	30.7% (194)	32.5% (902)	
Extremely satisfied	48.3% (35)	53.1% (1476)	
Getting along with household			
Very badly	2.3% (12)	0.4% (9)	<0.001
Badly	5.6% (30)	2.1% (50)	
Neither well nor badly	15.4% (82)	12.3% (291)	
Well	38.6% (205)	37.6% (8880	
Very well	38.0% (202)	47.5% (1122)	
Change in contact outside bubble			
Decreased	37.1% (234)	30.3% (837)	0.002
Stayed the same or increased	62.9% (396)	69.7% (1924)	
Feeling lonely or isolated			
All of the time	6.7% (42)	1.5% (43)	<0.001
Most of the time	9.4% (59)	4.6% (127)	
Some of the time	25.0% (158)	19.6% (544)	
A little of the time	30.6% (193)	28.4% (789)	
None of the time	28.4% (179)	45.9% (1275)	
Work			
Increased workload	25.9% (97)	19.6% (334)	0.019
Reduced paid hours of work	38.6% (144)	36.3% (620)	0.398
Employment terminated	5.2% (18)	6.1% (93)	0.553
Covid-19			
Tested for Covid-19			
Tested	6.0% (38)	3.3% (93)	<0.001

A greater proportion of those with a previous diagnosis of a mental illness reported increased workload compared to those without. There were no differences between the two groups in terms of reduced hours of paid work or the proportion who had had employment terminated.

Although numbers were low overall, those with a previous diagnosis of a mental illness reported higher rates of COVID-19 testing compared with those without such a diagnosis, with 6% having been tested for COVID-19. Numbers of confirmed positive tests were low; i.e. a total of 10 positive tests in the samples; this included six with mental illness and four without (Fisher’s exact, *p* = 0.004).

### Main sources of stress

Participants with a previous diagnosis of mental illness were more likely than those without to be concerned about both their own health (25.5% vs 15.4%, *p* < 0.001) and the health of others (40.7%, vs 32.0%, *p* < 0.001). They were also more likely to be concerned about finances (36.2% vs 29.7%, *p* = 0.001) and stress in relation to the wider consequences of COVID-19 (54.0% vs 47.6%, *p* = 0.004). There was no difference between the groups in concerns about employment (21% vs 20.1%, *p* = 0.583).

Only those with a previous diagnosis of mental illness were asked whether there had been a change in their mental health since the lockdown. Of these, 32% (197) reported their mental health had been worse or much worse than usual, 50% (316) the same and 18% (118) that it had been better or much better than usual. (One preferred not to answer.)

## Discussion

The aim of this study was to compare psychological outcomes, experiences and sources of stress during the New Zealand COVID-19 lockdown in people with and without a previous diagnosis of mental illness. The key findings were that those with a previous diagnosis of mental illness were at increased risk of psychological distress, anxiety, poor well-being, suicidality and increased alcohol consumption.

### Psychological outcomes

Participants with a history of mental illness reported significantly higher rates of detrimental psychological outcomes compared to those without a previous diagnosis. These findings are consistent with the emerging literature from general population (O’Connor et al., 2020; Robillard et al., 2020) and case–control studies conducted over a similar phase of the pandemic when lockdowns were first implemented (Hao et al., 2020; Iasevoli et al., 2020; Van Rheenen et al., 2020). While these studies utilised different methodologies, they also report a two to three times greater risk of moderate to severe anxiety and depressive symptoms in those with a mental health disorder (Iasevoli et al., 2020). Interestingly, these studies were from countries (China, Italy, the United Kingdom) which, at the time, had been significantly more impacted by the COVID-19 pandemic, with much higher rates of infection and mortality than were experienced in New Zealand.

Of course, higher rates of psychological distress, depression, anxiety, poor well-being and suicidality would be expected in people with mental illness compared to those without and cannot be attributed solely to reactions to the pandemic. Unfortunately, we do not have measures which allow direct pre-COVID comparisons in those with a mental health illness. However, in our earlier general population study (Every-Palmer et al., 2020), we noted that rates of psychological distress and poor well-being over the pandemic lockdown were well above baseline measures from past population surveys in New Zealand. In our study, female respondents reported higher rates of mental illness than males. This is likely a reflection of the majority of the identified mental illnesses being depression and anxiety with established female preponderance (Seedat et al., 2009).One in three of those in our study with a previous diagnosis of mental illness subjectively rated their mental health as worse than usual over the lockdown.

There was a small difference between those with and without previous diagnosis of mental illness in terms of increased alcohol use, with 30% and 24% of respondents, respectively, reporting this. This finding is consistent with that from an Australian study, which reported higher rates of increased drinking since the pandemic in those with a mood disorder compared with those without a mental health disorder (Van Rheenen et al., 2020).

While the findings above are important, it is also notable that the majority of respondents with or without a previous diagnosis of mental illness did *not* report detrimental psychological outcomes. In our study, 57% of those with and 81% of those without a previous diagnosis of mental illness did not report moderate to severe psychological distress, and 75% of those with and 91% of those without a previous diagnosis of mental illness did not report significant anxiety. In addition, half the participants who had been diagnosed with a mental health disorder reported their mental health was the same as usual, and one in six said it improved over lockdown. This reflects previous findings from populations exposed to potentially traumatising events, that, despite considerable challenges, the most common responses are of ‘resilience’ or ‘recovery’ (initial increase in distress followed by recovery) (Galatzer-Levy et al., 2018). It also highlights the importance of investigating both the positive and negative consequences of the pandemic (Jenkins et al., 2021).

### Sources of stress for those with a previous diagnosis of mental illness

It has been suggested that a constellation of stressors associated with the COVID-19 pandemic may contribute to poor psychological well-being (Holmes et al., 2020). These stressors include financial and employment uncertainty, reduced social contacts, diminished access to mental health supports and services, and worries about the risks of infection. Although the entire population may be exposed to some of these stressors, the impact on those with and without histories of mental illness may be different. Our study attempted to explore some of these issues.

As reported previously, and confirmed by our current findings, concerns about the health of family and friends are more prevalent than concerns about people’s own health. We found that people with a previous diagnosis of mental illness were more worried – both about their own and other people’s health. They were also at greater risk of having been tested, and testing positive, for COVID-19. This heightened risk of infection may relate to our finding of increased health vulnerabilities to COVID-19 in people with a previous diagnosis of mental illnesses. As we noted in the introduction, people with pre-existing mental health disorders, particularly severe mental illnesses, are at increased risk of contracting COVID-19 and of adverse morbidity and mortality associated with this (Taquet et al., 2021; Yang et al., 2020). These higher risks are likely to be due to medical and socioeconomic factors and make a strong ethical case for this group to be prioritised in vaccination strategies (implemented in some countries such as the United Kingdom) and for those providing care to provide clear information of the benefits and risk of vaccination (Mazereel et al., 2021).

Our findings support those from a UK study (O’Connor et al., 2020) and reflect concerns previously highlighted about the impacts of the pandemic on the social networks of people with mental health disorders (Yao et al., 2020). Participants with a previous diagnosis of mental illness reported less satisfaction with their lockdown living arrangements. They also reported reduced social contacts with family and friends and increased loneliness. This is an important finding in view of the well-established evidence that social isolation and loneliness are risk factors for detrimental psychological effects (Cacioppo et al., 2006; Lee et al., 2021). In terms of the economic impacts of the lockdown, our participants with a previous diagnosis of mental illness were more likely to report concern about finances. However, contrary to other studies, people in our study with a previous diagnosis of mental illness were not at greater risk of their employment being terminated or reduced (Robillard et al., 2020; Van Rheenen et al., 2020) and did not report stress about employment. It is possible that this may be explained by the NZ Government’s implementation of COVID-19 wage subsidies to cover lost income over the lockdown.

### Implications

The mental health of those with previous diagnosis of mental illness was particularly affected by the COVID-19 lockdown in New Zealand. This group needs to be prioritised to ensure they receive support, and this support should be targeted to address appropriate needs. While heightened in those with a previous diagnosis of mental illness, all respondents reported concern about the safety of themselves, their family and their friends, highlighting how crucial this concern is for most people. This finding underpins the established evidence for the importance of feeling safe and its association with resilient outcomes after disasters and previous epidemics (Hobfoll et al., 2015). Our findings also emphasise the importance of social connections for protecting against the potentially detrimental psychological impacts of public health restrictions. Again, there is established evidence of the benefits of maintaining social connections for all, but it should be a particular priority for those with a mental illness.

The longitudinal impacts of the COVID-19 pandemic for people with a pre-existing mental illness remain an important area for study, with several centres conducting ongoing research (Moore et al., 2020) (www.covidsocialstudy.org/) (Iob et al., 2020). It is important that this work continues to inform understanding of the issues people with mental health disorders face, and what is helpful for them.

### Strengths and limitations

Like all survey-based research, our study has some limitations. Outcomes were participants’ self-reports of a previous diagnosis of mental illness and subjective reports of their experiences and emotions. While these are not equivalent to a structured diagnostic interview (Levis et al., 2019), they do allow for comment on levels of distress. The measures used were quantitative, and further studies would benefit from a qualitative component to add richness to the findings.

There may have been some selection bias in our samples since respondents needed access to a computer or Internet-connected mobile phone to complete the survey. In addition, people for whom the topic of well-being had particular salience (perhaps because they were struggling) may have been more inclined to participate. The data were collected cross-sectionally and our analysis was limited by the lack of pre-COVID-19 benchmarks, which means we cannot be certain our results were solely caused by the pandemic and/or the lockdown. However, we were able to adjust for some potential confounders that might explain differences between those with or without a mental illness. This included adjustment for health status, living arrangements and other determinants of health (e.g. income and employment status). However, in a cross-sectional study, there is limited ability to determine directionality of some effects (e.g. the impact of loneliness on outcomes) when these are measured contemporaneously, thus we deliberately excluded adjustment for covariates that reflected mental health status after the start of lockdown. A more focussed study would be required to appropriately consider how the impact of prior mental health on these outcomes could be mediated by factors such as lack of support networks.

Other limitations relate to the small numbers in some of the mental health diagnostic groupings, which meant that finer-grained analyses were not possible.

A major strength of the study is that it is the first, of which we are aware, to examine the psychosocial outcomes of the lockdown in New Zealand in those with a previous diagnosis of mental illness. It is also the first wave in a longitudinal project, which aims to repeat the survey after 1 year to allow for examination of long-term impacts. Other strengths are that the survey questionnaire was peer reviewed during development and then pre-tested on a sample of members of the general public and used validated outcome measures wherever possible.

## Conclusion

Our study reports on the mental health and well-being of people with and without a previous diagnosis of mental illness during the New Zealand lockdown, early in the COVID-19 pandemic. While over half of those with a previous diagnosis of mental illness coped well, others did not. Those with a previous diagnosis of mental illness were at increased risk of psychological distress, anxiety and poor well-being compared with those without such a diagnosis. We suggest that government and health providers need to recognise the challenges this group face in times of pandemics and implement appropriate support for them. We would also emphasise the need for ongoing collection of robust mental health data to guide these approaches.

## Supplemental Material

sj-docx-1-anp-10.1177_00048674211034317 – Supplemental material for Psychological distress, loneliness, alcohol use and suicidality in New Zealanders with mental illness during a strict COVID-19 lockdown Click here for additional data file. Supplemental material, sj-docx-1-anp-10.1177_00048674211034317 for Psychological distress, loneliness, alcohol use and suicidality in New Zealanders with mental illness during a strict COVID-19 lockdown by Caroline Bell, Jonathan Williman, Ben Beaglehole, James Stanley, Matthew Jenkins, Philip Gendall, Charlene Rapsey and Susanna Every-Palmer in Australian & New Zealand Journal of Psychiatry
