# Supplementary material for: Psychological distress, loneliness, alcohol use and suicidality in New Zealanders with mental illness during a strict COVID-19 lockdown
Source: Aust N Z J Psychiatry. 2021 Jul 27;56(7):800–10. doi: 10.1177/00048674211034317 (PMC9218412; doi:10.1177/00048674211034317)
Supplement: sj-docx-1-anp-10.1177_00048674211034317 – Supplemental material for Psychological distress, loneliness, alcohol use and suicidality in New Zealanders with mental illness during a strict COVID-19 lockdown [file sj-docx-1-anp-10.1177_00048674211034317.docx]

Unadjusted

Demographics

Fully adjusted

K10 High/Very high

GAD-7 Moderate/Sever

WHO <13

Suicide Contemplate

Suicide Plans

Alcohol increase

Dynata

Ministries

Dynata

Ministries

Dynata

Ministries

1

2

3

1

3

5

1.0

1.5

2.0

2.5

1

3

10

1

10

100

1.0

1.2

1.4

1.6

Dataset

Risk ratio (95% CI)

Fig 1: Unadjusted and adjusted risk ratios for psychological distress, anxiety, poor wellbeing and increase in suicidality and alcohol use between those with versus without a previous history of mental illness. Points and vertical lines represent risk ratios and 95% confidence intervals respectively. Models are unadjusted, or adjusted for demographics (age, gender and ethnicity) or fully adjusted (age, gender, ethnicity, income, smoking status, employment, living alone, health vulnerability, prior mental health and prior exposure to a traumatic event). Participants in samples one and two come from the Dynata and NZ Ministries of Health and Justice datasets respectively.

Table s1: Living circumstances, social connections, workload, and COVID-19 testing. Sample one (Dynata) dataset.

| **Living circumstances, social connections, work demand, and COVID-19 testing** | **Previous diagnosis of mental illness** | **No previous diagnosis of mental illness** | ***p*** |
| --- | --- | --- | --- |
| **Living circumstances** |  |  |  |
| *Living situation* |  |  |  |
| Living alone | 16.5% (62) | 12.9% (205) | 0.246 |
| With one adult | 28.3% (106) | 30.1% (479) |  |
| With other adults | 23.7% (89) | 22.8% (362) |  |
| With children | 31.5% (118) | 34.3% (545) |  |
| *Satisfaction with ‘bubble’* |  |  |  |
| Extremely dissatisfied | 5.3% (20) | 3.2% (51) | <0.001 |
| Dissatisfied | 7.7% (29) | 2.1% (34) |  |
| Neither satisfied nor dissatisfied | 13.1% (49) | 10.9% (174) |  |
| Satisfied | 29.9% (112) | 35.5% (565) |  |
| Extremely satisfied | 44.0% (165) | 48.2% (767) |  |
| *Getting along with household* |  |  |  |
| Very badly | 3.5% (11) | 0.6% (9) | <0.001 |
| Badly | 7.4% (23) | 2.8% (39) |  |
| Neither well nor badly | 20.2% (63) | 16.5% (229) |  |
| Well | 37.5% (117) | 39.8% (552) |  |
| Very well | 31.4% (98) | 40.2% (557) |  |
| *Change in contact outside bubble* |  |  |  |
| Decreased | 41.8% (156) | 32.9% (520) | 0.003 |
| *Feeling lonely or isolated* | 29.8% (111) | 36.8% (582) |  |
| All of the time | 9.1% (34) | 2.1% (33) | <0.001 |
| Most of the time | 13.9% (52) | 6.5% (103) |  |
| Some of the time | 27.0% (101) | 22.7% (361) |  |
| A little of the time | 24.6% (92) | 26.6% (423) |  |
| None of the time | 25.4% (95) | 42.2% (671) |  |
| **Work** |  |  |  |
| *Increased workload* | 29.5% (59) | 19.0% (179) | 0.003 |
| .. *Reduced paid hours of work* | 46.2% (92) | 39.0% (367) | 0.057 |
| *Employment terminated* | 7.0% (13) | 9.1% (83) | 0.343 |
| **Covid-19** |  |  |  |
| *Tested for Covid-19* |  |  |  |
| Tested | 6.2% (27) | 3.9% (62) | <0.001 |

Table s2: Living circumstances, social connections, workload, and COVID-19 testing. Sample two (NZ Ministries of Health and Justice) dataset.

| **Living circumstances, social connections, work demand, and COVID-19 testing** | **Previous diagnosis of mental illness** | **No previous diagnosis of mental illness** | ***p*** |
| --- | --- | --- | --- |
| **Living circumstances** |  |  |  |
| *Living situation* |  |  |  |
| Living alone | 14.8% (38) | 18.0% (213) | 0.014 |
| With one adult | 30.4% (78) | 37.0% (438) |  |
| With other adults | 20.6% (53) | 14.0% (166) |  |
| With children | 34.2% (88) | 30.9% (366) |  |
| *Satisfaction with ‘bubble’* |  |  |  |
| Extremely dissatisfied | 4.3% (11) | 5.4% (64) | 0.300 |
| Dissatisfied | 1.9% (5) | 1.3% (15) |  |
| Neither satisfied nor dissatisfied | 7.4% (19) | 5.2% (62) |  |
| Satisfied | 31.9% (82) | 28.4% (337) |  |
| Extremely satisfied | 54.5% (140) | 59.7% (709) |  |
| *Getting along with household* |  |  |  |
| Very badly | 0.5% (1) | 0.0% (0) | 0.003 |
| Badly | 3.2% (7) | 1.1% (11) |  |
| Neither well nor badly | 8.7% (19) | 6.4% (62) |  |
| Well | 40.2% (88) | 34.5% (336) |  |
| Very well | 47.5% (104) | 58.0% (565) |  |
| *Change in contact outside bubble* |  |  |  |
| Decreased | 30.4% (78) | 26.8% (317) | 0.426 |
| *Feeling lonely or isolated* | 33.5% (86) | 37.1% (438) |  |
| All of the time | 3.1% (8) | 0.8% (10) | <0.001 |
| Most of the time | 2.7% (7) | 2.0% (24) |  |
| Some of the time | 22.2% (57) | 15.4% (183) |  |
| A little of the time | 39.3% (101) | 30.8% (366) |  |
| None of the time | 32.7% (84) | 50.9% (604) |  |
| **Work** |  |  |  |
| *Increased workload* | 21.8% (38) | 20.2% (155) | 0.888 |
| .. *Reduced paid hours of work* | 29.9% (52) | 33.0% (253) | 0.430 |
| *Employment terminated* | 3.2% (5) | 1.6% (10) | 0.200 |
| **Covid-19** |  |  |  |
| *Tested for Covid-19* |  |  |  |
| Tested | 4.3% (11) | 2.6% (31) | 0.247 |

Other

COVID19

Employment

Finances

Other health

Own health

0.8

0.9

1.0

1.1

1.2

1.3

1.5

1.7

2.0

2.5

Risk ratio (95% CI)

Source of stress

Dataset

Dynata

Minisitries

Figure 2: Unadjusted risk ratios for sources of stress between those with versus without a previous history of mental illness. Points and horizontal lines represent risk ratios and 95% confidence intervals respectively. The vertical dashed line represents the line of no effect. Participants in samples one and two come from the Dynata and NZ Ministries of Health and Justice datasets respectively.
